# Mathematical Modeling of Interacting Glucose-Sensing Mechanisms and Electrical Activity Underlying Glucagon-Like Peptide 1 Secretion

**DOI:** 10.1371/journal.pcbi.1004600

**Published:** 2015-12-02

**Authors:** Michela Riz, Morten Gram Pedersen

**Affiliations:** Department of Information Engineering, University of Padua, Padua, Italy; Johns Hopkins University, UNITED STATES

## Abstract

Intestinal L-cells sense glucose and other nutrients, and in response release glucagon-like peptide 1 (GLP-1), peptide YY and other hormones with anti-diabetic and weight-reducing effects. The stimulus-secretion pathway in L-cells is still poorly understood, although it is known that GLP-1 secreting cells use sodium-glucose co-transporters (SGLT) and ATP-sensitive K^+^-channels (K(ATP)-channels) to sense intestinal glucose levels. Electrical activity then transduces glucose sensing to Ca^2+^-stimulated exocytosis. This particular glucose-sensing arrangement with glucose triggering both a depolarizing SGLT current as well as leading to closure of the hyperpolarizing K(ATP) current is of more general interest for our understanding of glucose-sensing cells. To dissect the interactions of these two glucose-sensing mechanisms, we build a mathematical model of electrical activity underlying GLP-1 secretion. Two sets of model parameters are presented: one set represents primary mouse colonic L-cells; the other set is based on data from the GLP-1 secreting GLUTag cell line. The model is then used to obtain insight into the differences in glucose-sensing between primary L-cells and GLUTag cells. Our results illuminate how the two glucose-sensing mechanisms interact, and suggest that the depolarizing effect of SGLT currents is modulated by K(ATP)-channel activity. Based on our simulations, we propose that primary L-cells encode the glucose signal as changes in action potential amplitude, whereas GLUTag cells rely mainly on frequency modulation. The model should be useful for further basic, pharmacological and theoretical investigations of the cellular signals underlying endogenous GLP-1 and peptide YY release.

## Author Summary

Metabolic diseases are to a great extent because of disturbances in hormone secretion. Endocrine cells releasing hormones with a role in metabolism typically possess a refined molecular system for nutrient sensing, which allows them to respond in an appropriate manner to changes in e.g. glucose levels. The gut is the largest endocrine organ of the human body due to a range of endocrine cells that are strategically located to sense nutrient levels in response to food intake. The intestinal L-cells secrete glucagon-like peptide 1 (GLP-1), peptide YY and other hormones with anti-diabetic and weight-reducing effects, but the stimulus-secretion cascade in L-cells is still only partly understood. Here we dissect glucose sensing underlying GLP-1 secretion using mathematical modeling of electrical activity in primary L-cells and the GLP-1 secreting GLUTag cell line. We cast new light on the differences in glucose-sensing between the two cell types, and we propose that primary L-cells encode the glucose signal as changes in action potential amplitude, whereas GLUTag cells rely mainly on frequency modulation. Our results should be of general interest for understanding glucose-sensing in various cell types.

## Introduction

Glucose sensing by a variety of specialized cells located, for example, in the pancreas [1], the brain [2] and the ingestive tract [3], plays a crucial role in the control of body weight and blood glucose levels, and dysfunctional glucose sensing is involved in the development of obesity and diabetes [2]. The various glucose-sensing cells rely on different molecular mechanisms for monitoring glucose levels. The prototype mechanism operating in pancreatic *β*-cells involves glucose-uptake by GLUT transporters and closure of ATP-sensitive potassium (K(ATP)-) channels, which leads to cell depolarization and action potential firing with subsequent insulin release [1]. However, for example the enteroendocrine L-cells use the electrogenic sodium glucose co-transporter 1 (SGLT1) to link glucose stimulus to electrical activity and secretion [4–9] with a possible minor role for K(ATP)-channels [4, 9]. Similarly, SGLTs are involved in glucose sensing in the hypothalamus [10], and play a role in pancreatic *α*-cells [11] in addition to K(ATP)-channels [1].

Glucagon-like peptide 1 (GLP-1) is an insulinotropic hormone released from intestinal L-cells in response to food ingestion [12]. It is, together with other hormones, responsible for the so-called incretin effect, i.e., the fact that glucose ingested orally elicits a greater insulin response than glucose administered intravenously, even when glucose concentrations in plasma are matched. In addition, GLP-1 inhibits glucagon secretion, slows gastric emptying, regulates appetite and food intake, stimulates *β*-cell neogenesis and proliferation, and promotes *β*-cell survival both *in vitro* and *in vivo* [12], and deficient incretin signalling has been suggested to be a major reason of insufficient insulin release and excessive glucagon release in type-2 diabetics [13].

The beneficial effects of GLP-1 have led to incretin-based therapies, and GLP-1 mimetics and inhibitors of GLP-1 degradation are already available [14]. Recently, alternative treatments, aiming at enhancing endogenous secretion from the intestinal L-cells directly, are under investigation [3, 15, 16]. However, the nutrient sensing mechanisms and the secretory pathways in L-cells remain still incompletely understood [17–19].

The GLP-1 secreting cell line GLUTag [20] has been widely used to obtain insight into the cellular mechanisms leading to GLP-1 release. GLUTag cells use the electrogenic SGLT1 [21] and K(ATP)-channels [22] to sense glucose. Electrical activity then promotes Ca^2+^ influx and release of GLP-1 [23]. Subsequent studies using transgenic mice with fluorescent L-cells [4] confirmed that primary L-cells rely on similar mechanisms to transduce glucose sensing to GLP-1 secretion [4, 17]. However, differences in the electrophysiological properties of GLUTag [23] and primary L-cells [24] have emerged, which could underlie the variation in secretory responses in GLUTag versus L-cells. In particular, primary L-cells appear to rely mainly on SGLT1 for glucose sensing, in contrast to GLUTag cells, which use both SGLT1 and K(ATP)-channels to transduce glucose stimuli to GLP-1 secretion [4–9, 21, 22].

Related to the relative roles of SGLT1 and K(ATP)-channels is the debate on how SGLT1 and GLUT2 glucose transporters contribute to glucose sensing in L-cells [8]. As mentioned above, the electrogenic SGLT1 transporters could directly induce electrical activity, whereas glucose entering via GLUT2 should be metabolized to increase the ATP levels and reduce K(ATP)-channel activity to promote action potential firing. SGLT1 transporters are located on the luminal, apical side of the L-cells, and are therefore exposed directly to glucose in the intestine [8]. In contrast, GLUT2 is located on the vascular side of the L-cells [8]. It has been suggested that GLUT2 can be inserted into the luminal membrane of enterocytes in response to glucose by a SGLT1-dependent mechanism [6, 25, 26], though other studies have cast doubt on this hypothesis [8]. Thus, a better understanding of the glucose-sensing mechanisms leading to electrical activity might also shed new light on the relative roles of SGLT1 and GLUT2 transport underlying GLP-1 secretion.

The subtle differences in ion channel characteristics between the two GLP-1 secreting cell types complicate intuitive reasoning on the interplay of the various currents underlying GLP-1 release. In this context, a mathematical model could be useful to get a deeper insight into the stimulus-secretion pathway. Mathematical modeling has been used to study glucose sensing in pancreatic *β*-cells [27–29] and *α*-cells [30–32], and we recently modelled human *β*-cells to investigate how species differences and cellular heterogeneity in electrophysiological properties are reflected in electrical activity [33–35].

Here, we present a mathematical model of electrical activity underlying GLP-1 release built directly from experimental data. A single model for primary L-cells and GLUTag cells is presented but with two sets of parameters to represent the two cell types. Thus, we investigate how the differences in ion channel characteristics translate into different electrophysiological responses in primary L-cells and GLUTag cells with particular focus on glucose sensing by SGLT1 and K(ATP)-channels. Further, we discuss how the simulations based on data from cultured cells can give insight into L-cells *in situ* with preserved physiological polarization.

## Results

### Glucose sensing mechanisms

Experimentally, it is possible to stimulate the two glucose-sensing mechanisms individually by using different sugar types. For example, alpha-methyl-D-glucopyranoside (*α*MG) is a non-metabolizable glucose analogue that is co-transported by SGLT1 and can depolarize the cell by a SGLT1-associated current without inducing K(ATP)-channel closure. Fructose, on the other hand, does not enter via SGLT1, but is metabolized and the resulting ATP increase closes K(ATP)-channels [21, 36]. We note that in contrast to *β*-cells, which utilize fructose poorly [37, 38] and are unresponsive to fructose alone [38, 39], GLUTag cells efficiently metabolize fructose [36], which triggers electrical activity [21]. Finally, a glucose stimulus might be sensed by the two different pathways simultaneously, and the model could help in differentiating the contribution of each pathway. Instead of fructose, the K(ATP)-channel blocker tolbutamide is commonly used to target K(ATP)-channels without affecting the SGLT1-associated current. Glucose-induced changes in K(ATP)-channel conductance, *g*_*K*(*ATP*)_, in physiological settings is the consequence of glucose transport, mainly via GLUT2 [7], and its subsequent metabolization.

To simplify the notation, in the following the term SGLT1-substrate will represent any substance that is cotransported by SGLT1 and induces the associated current. In the model described in the Methods, a SGLT1-substrate corresponds to the parameter *G*_SGLT1_, which represents the extracellular concentration of e.g. glucose or *α*MG. Physiologically, this would be the major glucose stimulus from the intestine, since SGLT1 is located on the luminal side of the L-cells and is pivotal for physiological GLP-1 secretion [6, 8, 9].

#### GLUTag cells

In GLUTag cells electrical activity was promoted by stimulation with glucose [21–23]; *α*MG [21], acting on SGLT1 only; or with tolbutamide [22] or fructose [21], which affect K(ATP)-channels only. In contrast to the primary L-cells, a glucokinase activator (GKA50) augmented GLP-1 secretion from GLUTag cells at 1 or 10 mM glucose [7]. These experiments showed that although both electrogenic SGLT1 uptake and sugar metabolism independently can trigger action potential firing in GLUTag cells, the two mechanisms interact and both play a direct role in glucose sensing in the cell line.

**Fig 1:**
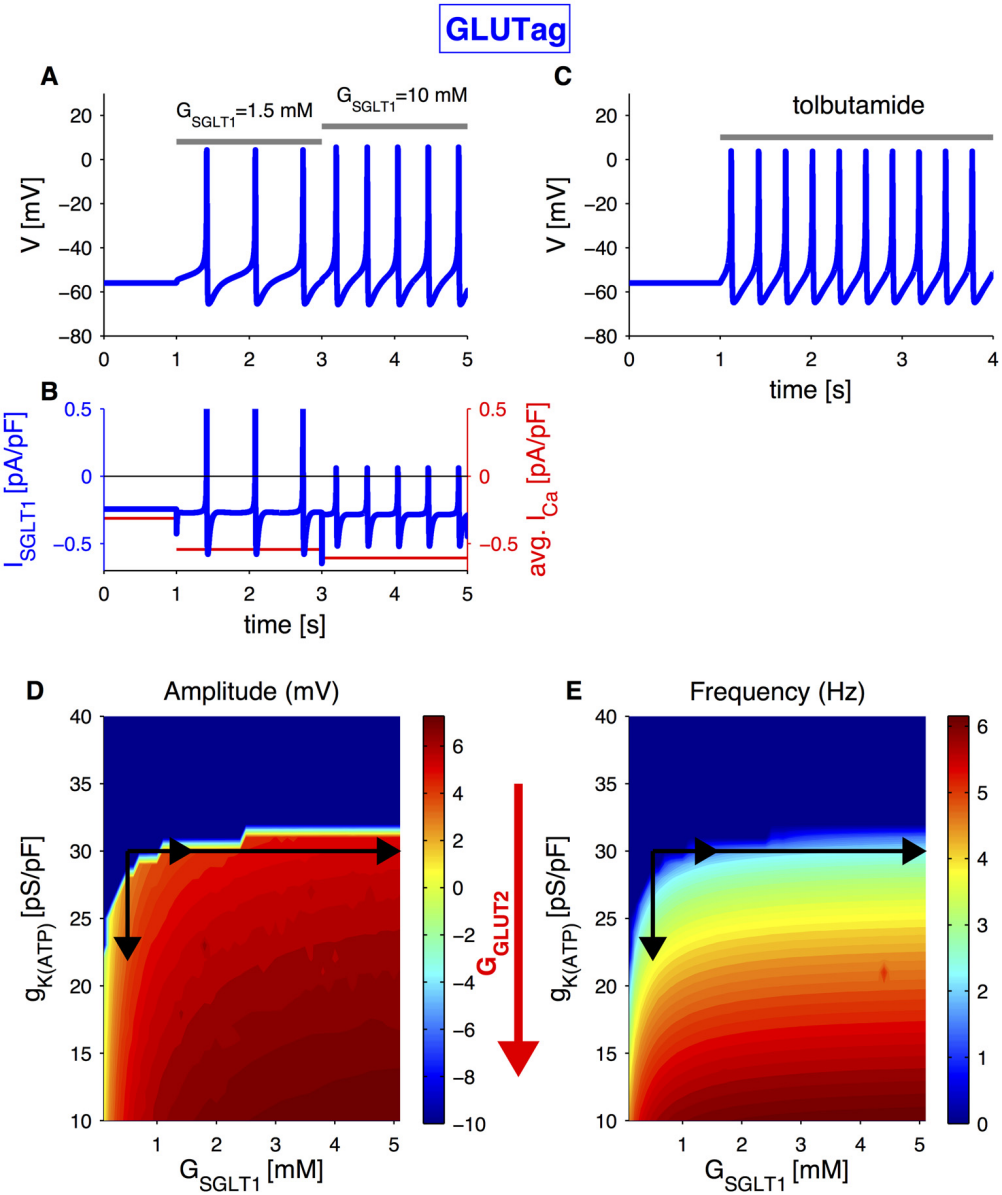
Simulated electrical activity in the GLUTag cell line. A: Simulation of electrical activity triggered by SGLT1-substrate uptake, corresponding to *α*MG application, with default parameters and extracellular SGLT1-substrate concentration, *G*_SGLT1_, changing from 0.5 mM to 1.5 mM and 10 mM as indicated. B: SGLT1-associated current (left axis) and average calcium current (red, right axis) corresponding to the simulation in panel A. C: Simulation of electrical activity triggered by K(ATP)-channel closure with default parameters and K(ATP)-channel conductance, *g*_*K*(*ATP*)_, changed from from 30 pS/pF to 23 pS/pF as indicated by the bar. D: Voltage peak amplitude as a function of *G*_SGLT1_ and *g*_*K*(*ATP*)_. E: Spiking frequency as a function of *G*_SGLT1_ and *g*_*K*(*ATP*)_. In panels D and E, the black arrows indicate the parameter changes in panels A, B and C. The red arrow indicates the effect of an increase in intracellular glucose concentration due to GLUT2 transport.

We investigated whether electrogenic glucose uptake alone can evoke electrical activity in the mathematical model of the GLUTag cell line. In order to simulate SGLT1-mediated glucose uptake, with no effect on metabolism, it is sufficient to change the extracellular SGLT1-substrate concentration *G*_SGLT1_ and keep the K(ATP)-conductance *g*_*K*(*ATP*)_ unchanged. At low *G*_SGLT1_ (0.5 mM), the cell is in a silent state, and an elevation of *G*_SGLT1_ (1.5 mM or 10 mM) induces an inward transport of the SGLT1-substrate that generates a fast increase in the associated inward current, which is sufficient to depolarize the cell and initialize electrical activity (Fig 1A and 1B). The average calcium current (Fig 1B, red), a rough measure of GLP-1 secretion, increases significantly once the cell is electrically active, and Ca^2+^ influx augments further when spiking frequency increases as a consequence of *G*_SGLT1_ elevation (Fig 1A).

The model also reproduces the induction of electrical activity as a consequence of K(ATP)-channel block in response to tolbutamide [22] or fructose [21] (Fig 1C). In this case, the reduction in the outward potassium current is sufficient to allow depolarization and electrical activity.

In the simulations above, only one possible glucose-sensing mechanism is involved, i.e., we varied either the extracellular SGLT1-substrate concentration, having an effect on the SGLT1 current, or *g*_*K*(*ATP*)_, corresponding to the closure/opening of K(ATP)-channels as a consequence of glucose transport, mainly via GLUT2 [7]. However, to better understand the interaction between the two glucose-sensing mechanisms, the model response should be analysed by varying the two parameters simultaneously. Different combinations of the two parameters lead to different electrical activity responses, which can be characterized by the peak value of the membrane potential during spiking activity and the spiking frequency. The results are shown in Fig 1D for peak membrane potential and in Fig 1E for the frequency; the blue region of zero frequency corresponds to parameters where the cell is electrically silent. Furthermore, it can be seen that action potential amplitude is almost constant once the cell is electrically active, while firing frequency can be modulated by different combinations of *G*_SGLT1_ and *g*_*K*(*ATP*)_. Thus, the model suggests that GLUTag cells encode glucose sensing in the frequency, not the amplitude, of electrical activity. Experimental data support this notion [22].

Fig 1D and 1E becomes an useful tool to understand the simulations of electrical activity in GLUTag cells (Fig 1A and 1C). A cell in the silent state can become active as a result of an increase in extracellular SGLT1-substrate, which corresponds to the rightwards arrows in Fig 1D and 1E. Given the high affinity of SGLT1 to its substrate, a higher concentration would not result in a significant effect on electrical activity (see Fig 1D and 1E). Alternatively, the cell might become active as a result of K(ATP)-channel closure, which is represented by a downwards arrow in Fig 1D and 1E. A further decrease in *g*_*K*(*ATP*)_ would result in an increase of both spiking amplitude and frequency, supporting the hypothesis of K(ATP)-channels playing a role in setting GLUTag excitability.

**Fig 2:**
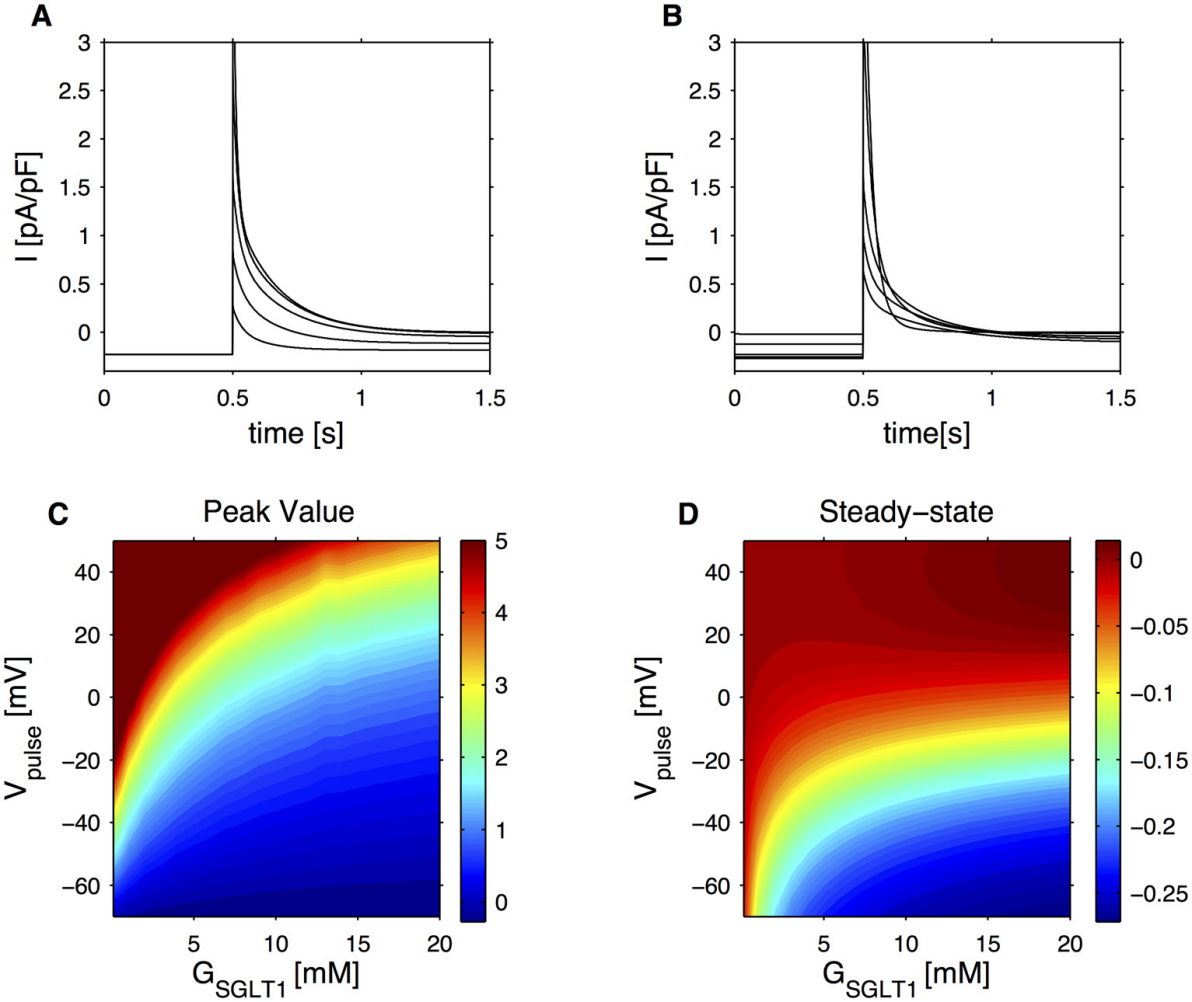
Model characterization of SGLT1-associated currents during voltage clamp as a function of SGLT1-substrate concentration, *G*_SGLT1_, and voltage pulse, *V*_pulse_ and parameters as for GLUTag model. The voltage-clamp protocol consisted in applying 1 s depolarization at *t* = 0.5 s from a holding potential of -70 mV. A: Simulated SGLT1-associated current in response to different voltage pulses (*V*_pulse_ = −50, −30, −10, 10, 30 mV) and constant *G*_SGLT1_ = 5 mM. B: Simulated SGLT1-associated current in response to a voltage pulse (*V*_pulse_ = −10 mV) and different *G*_SGLT1_ = 0.1, 1, 5, 10, 20 mM. C: Simulated peak SGLT1-associated current in response to voltage pulses as a function of *G*_SGLT1_ and *V*_pulse_. D: Simulated steady-state SGLT1-associated current in response to voltage pulses as a function of *G*_SGLT1_ and *V*_pulse_.

The simulated SGLT1-associated current becomes positive during the action potentials (Fig 1B), and therefore contributes to cell repolarization. This twofold role of the SGLT1-associated current, the depolarization effect to initiate the action potential and the repolarizing effect to terminate it, was further analysed with the model. In response to depolarizing pulses, the SGLT1 co-transporter generates a fast transient outward current followed by a sustained inward current, whose magnitude depends both on the voltage of the pulse (Fig 2A) and on extracellular SGLT1-substrate concentration (Fig 2B). At low SGLT1-substrate concentrations, the simulated transient outward current is bigger for more positive pulse potentials (*V*_pulse_), while the inward current current becomes smaller with increasing *V*_pulse_. As the SGLT1-substrate concentration increases, the transient outward current decreases, eventually becoming negligible, whereas the steady-state inward current increases with high affinity for the substrate (see Fig 2C and 2D). Thus, the effects of increasing *G*_SGLT1_ are different from augmenting the number of transporters (*n* in the model), which would increase the size of both the transient and sustained currents. As a consequence, at high SGLT1-substrate concentrations the model cell can initiate electrical activity thanks to the increased inward current, and the action potentials can reach slightly higher values because of the reduced outward current (see Fig 1A and 1B). These aspects are discussed in greater details below.

**Fig 3:**
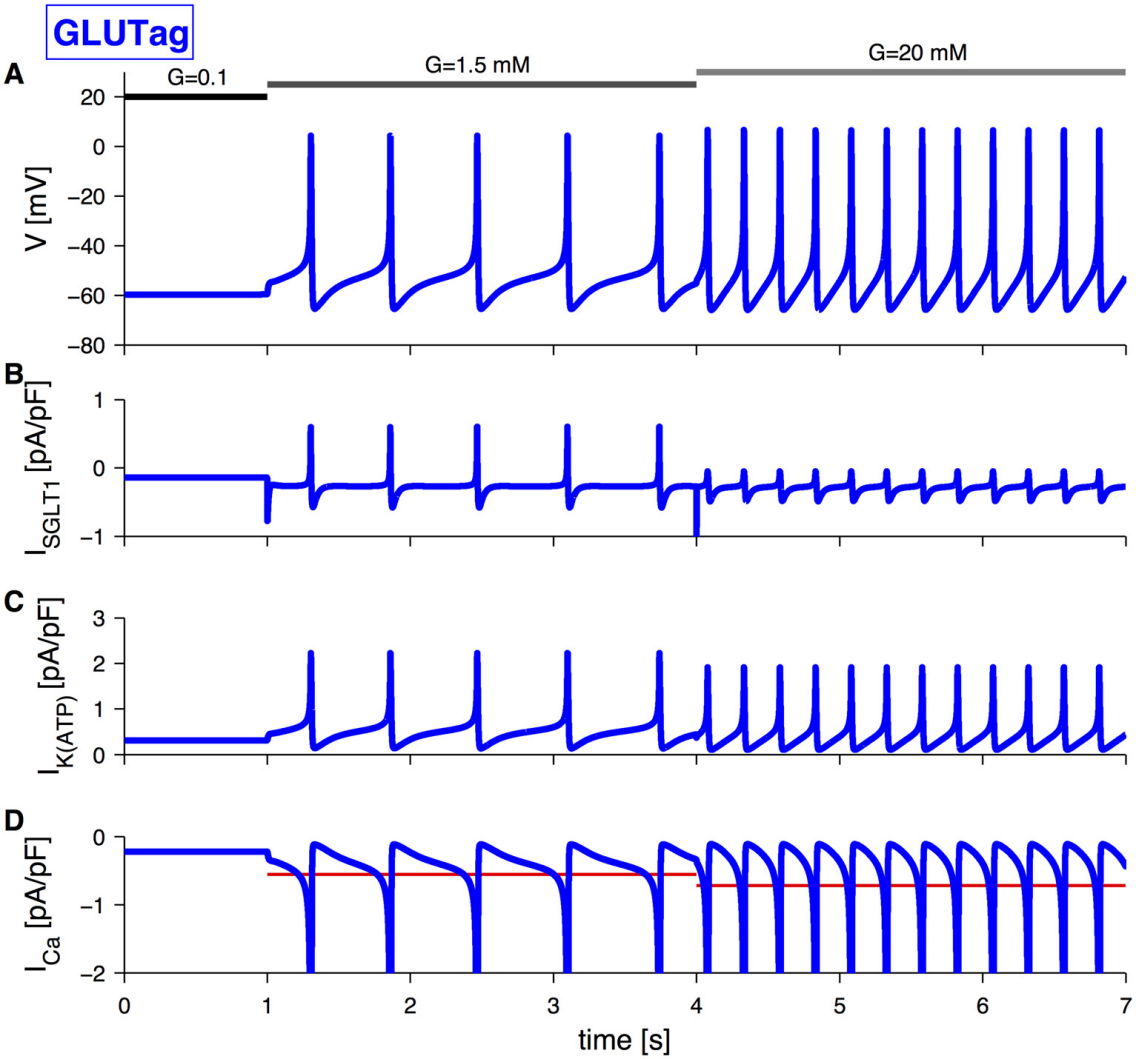
Effect of stimulation with glucose at different concentrations (indicated by grey bars) on GLUTag electrical activity. The stimulation with 1.5 mM glucose was simulated by changing extracellular SGLT1-substrate concentration, *G*_SGLT1_, from 0.1 mM to 1.5 mM, while *g*_*K*(*ATP*)_ remained unchanged from its default value. Subsequent 20 mM glucose application was simulated by changing *G*_SGLT1_ from 1.5 mM to 20 mM, and *g*_*K*(*ATP*)_ from 30 pS/pF to 25 pS/pF. A: Simulation of electrical activity triggered by different glucose concentrations. B: SGLT1-associated current corresponding to the simulation in panel A. C: K(ATP)-current corresponding to the simulation in panel A. D: Calcium current (blue) and its average (red) corresponding to the simulation in panel A.

A glucose stimulation would have an effect on both the K(ATP)-conductance and the SGLT1-mediated current, depending on its concentration. In particular, the ability of GLUTag cells to sense low concentrations of sugars might be attributable to the SGLT1-associated current, given the high glucose affinity of SGLT1, which in the model corresponds to a change in *G*_SGLT1_ with unchanged *g*_*K*(*ATP*)_. At higher glucose concentrations (>5 mM), glucose, transported via GLUT2, could have an effect on metabolism and closure of K(ATP)-channels, besides an increase in the SGLT1-associated current. The resulting membrane potential simulation is shown in Fig 3. The higher glucose concentration resulted in a small increase in the peak of the action potential and a greater increase in spiking frequency. The slightly increased peak amplitude and accelerated frequency are due to a combination of the closure of K(ATP)-channels (Fig 3C) and a reduction in the transient outward SGLT1-current (Fig 3B), given its twofold role explained above (Fig 2). Electrical activity increases calcium influx, and at higher glucose concentration a further increase in the average calcium current is visible (Fig 3D).

#### Primary L-cells

**Fig 4:**
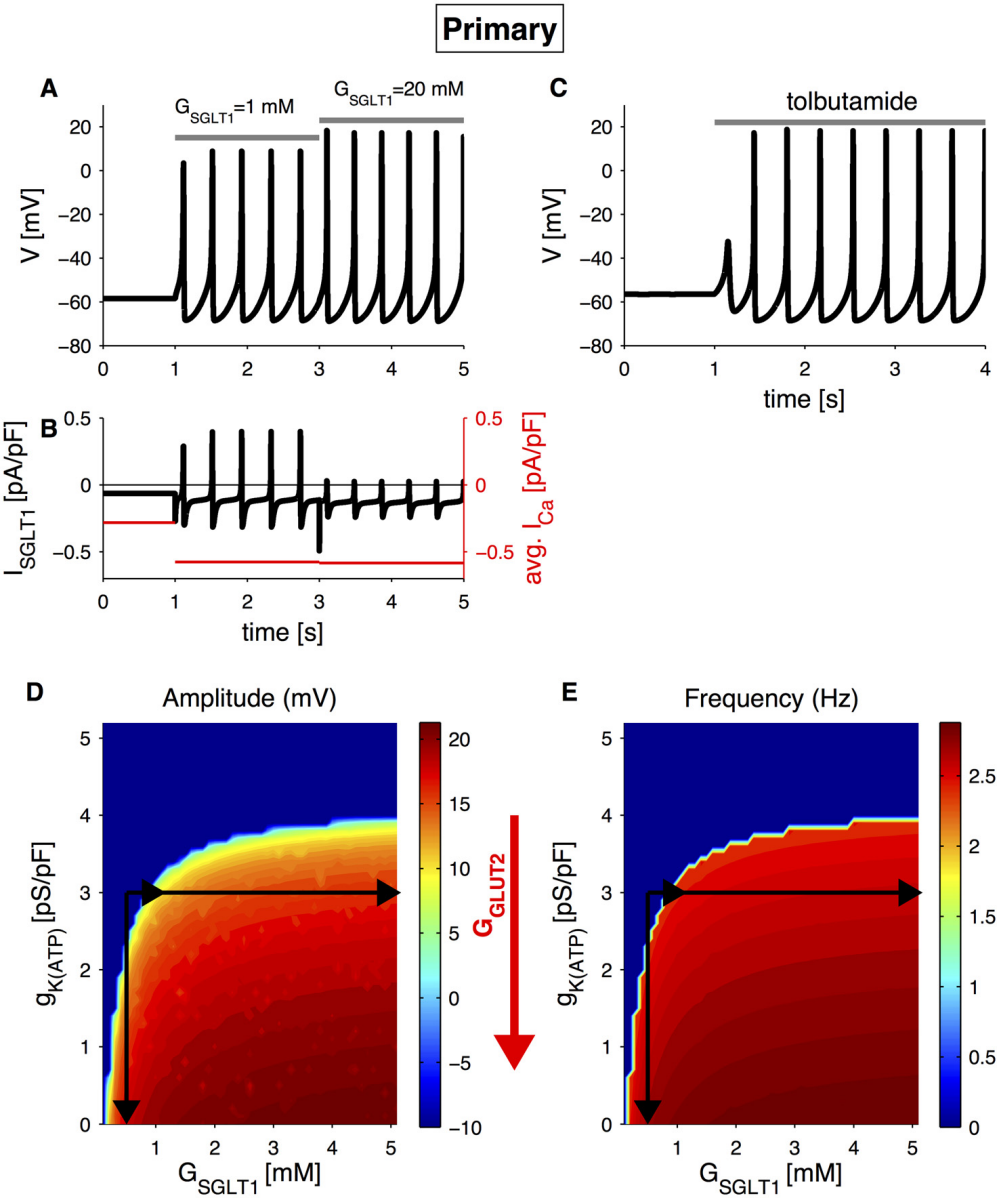
Simulated electrical activity in primary L-cells. A: simulation of electrical activity triggered by SGLT1-substrate uptake, corresponding to *α*MG application, in primary L-cells with default parameters and extracellular SGLT1-substrate, *G*_SGLT1_, changing from 0.1 mM to 1 mM and 20 mM as indicated. B: SGLT1-associated current (left axis) and average calcium current (red, right axis) corresponding to the simulation in panel A. C: Simulation of electrical activity triggered by the K(ATP)-channel blocker tolbutamide. Default parameters and K(ATP)-channel conductance, *g*_*K*(*ATP*)_, changing from from 3 pS/pF to 0 pS/pF. Grey bars indicate application of the substances. D: Voltage peak as a function of *G*_SGLT1_ and *g*_*K*(*ATP*)_. E: Spiking frequency as a function of *G*_SGLT1_ and *g*_*K*(*ATP*)_. In panels D and E, the black arrows indicate the parameter changes in panels A, B and C. The red arrow indicates the effect of an increase in intracellular glucose concentration due to GLUT2 transport.

As for the GLUTag cell line, similar simulations can be performed to analyse the contribution of the two sensing mechanisms in primary L-cells. To our knowledge, no experimental data on electrical activity with *α*MG, acting on SGLT1 transport alone, are available in the literature. However, with the mathematical model, we can now test directly whether electrogenic glucose uptake is sufficient to trigger electrical activity in primary L-cells. Similarly to the GLUTag cells, at low *G*_SGLT1_ (0.1 mM), the cell is in a silent state, and an elevation of *G*_SGLT1_ (1 mM or 20 mM) generates an increase in the SGLT1-associated inward current, causing cell depolarization and electrical activity (Fig 4A and 4B). A further increase in *G*_SGLT1_ does not affect electrical activity significantly because of the high affinity of SGLT1 to its substrate. Similarly, the average calcium current increases when the simulated cell becomes electrically active, but it is virtually unchanged when *G*_SGLT1_ is further elevated (Fig 4B). These simulations correspond to the physiological in vivo setting, where glucose enters from the lumen mainly via SGLT1 [5, 6, 8, 9]. Interestingly, glucose is more efficient that *α*MG in stimulating GLP-1 secretion from the perfused rat intestine [9], suggesting that glucose physiologically has additional effects on L-cells besides increasing the SGLT1 current. These additional mechanisms, which were not included in the present version of the model, appear not to be operating in cultured mouse L-cells, since glucose and *α*MG stimulate secretion similarly and with high affinity in these cells [4].

The model also reproduces the induction of electrical activity by the K(ATP)-channel antagonist tolbutamide [17] (Fig 4C). However, it is worth noting that the starting point for the primary L-cells corresponds to *g*_*K*(*ATP*)_ = 3 pS/pF, which means that on average only a single K(ATP) channel is open [40]. In contrast, the GLUTag cells have a ten-fold higher K(ATP) conductance, which might explain how stimulated metabolism by fructose [21] or glucokinase activators [7] can have an effect in GLUTag cells but not in primary L-cells: in the latter almost all K(ATP)-channels are already closed and further physiological inhibition is therefore not possible. Nonetheless, pharmacological closure of K(ATP)-channels by tolbutamide can trigger electrical activity and GLP-1 secretion [4, 9, 17], and our model shows that although the exogenous K(ATP)-channels activity is very low, a further reduction is sufficient to allow electrical activity.

The simulated electrical responses are summarized in Fig 4C and 4D, showing voltage peak and frequency, respectively, as a function of *G*_SGLT1_ and *g*_*K*(*ATP*)_. Similarly to the GLUTag cell line, the blue region of zero frequency is where the cell is electrically silent. In the area with action potential firing, the electrical activity changes by different combinations of *G*_SGLT1_ and *g*_*K*(*ATP*)_. In contrast to an active GLUTag cell, whose frequency can be finely modulated mainly by changing *g*_*K*(*ATP*)_ in the presence of *G*_SGLT1_, in the primary L-cell model the spiking frequency is only slightly affected by further changes in the parameters. Action potential amplitude can be increased by different combinations of *G*_SGLT1_ and *g*_*K*(*ATP*)_. The increase is bigger (∼10 mV) compared to the one obtain in the GLUTag model (∼3 mV) with similar parameter changes. Thus, the model suggests that primary L-cells use action potential amplitude rather than frequency to transduce glucose-sensing. The simulations show that average Ca^2+^ influx is almost unchanged by modifications in action potential amplitude. Thus, average Ca^2+^ influx may not be a good measurement of secretion in primary L-cells if exocytosis is controlled by local Ca^2+^ elevations. Moreover, additional mechanisms operating downstream of Ca^2+^ influx may underlie increased secretory responses to high glucose concentrations. Further studies should investigate these aspects, as has been done e.g. for pancreatic *α*-cells [32, 41].

### Ionic mechanisms of action potential generation

**Fig 5:**
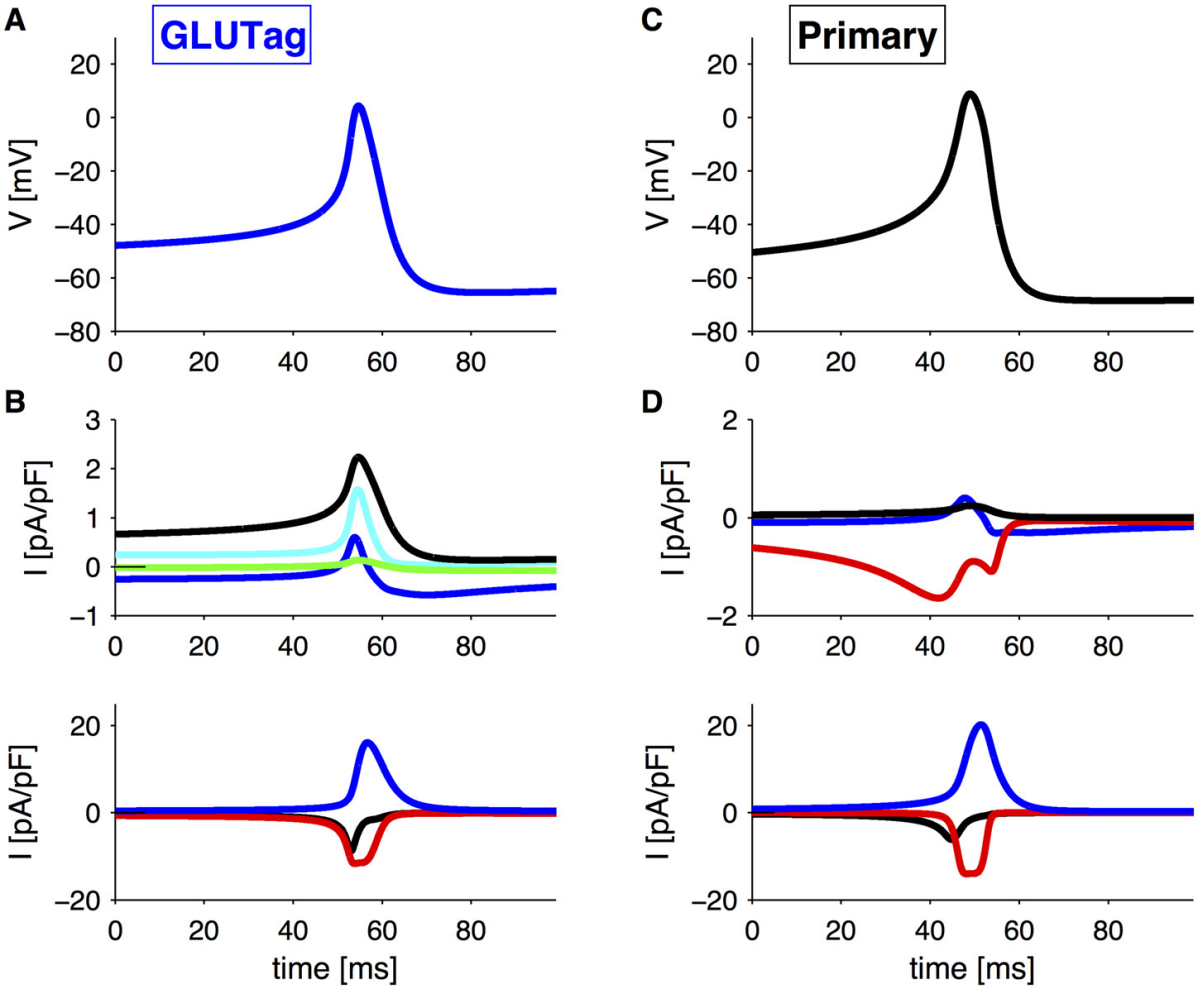
Membrane currents during a single action potential. A: Zoom on an action potential in GLUTag cells from Fig 1 with *G*_SGLT1_ = 1.5 mM. B: Membrane currents during the action potential in panel A. The currents have for clarity been divided according to their amplitude. Upper panel: *I*_*SGLT*1_ (blue), *I*_*K*(*ATP*)_ (black), *I*_*KA*_ (cyan), *I*_*K*,*hyper*_ (green). Lower panel: *I*_*Na*_ (black), *I*_*CaHV A*_ (red), *I*_*Kv*_ (blue). C: Zoom on an action potential in primary L-cells from Fig 4 with *G*_SGLT1_ = 1 mM. D: Membrane currents during the action potential in panel C. Upper panel: *I*_*SGLT*1_ (blue), *I*_*K*(*ATP*)_ (black), *I*_*CaT*_ (red). Lower panel: *I*_*Na*_ (black), *I*_*CaHV A*_ (red), *I*_*Kv*_ (blue).

To investigate more closely how the different membrane currents contribute to create and shape action potentials in the two cell types, we plotted the different currents during an action potential. In GLUTag cells (Fig 5A and 5B), the sustained, inward SGLT1-current and a small Ca^2+^ current depolarize the membrane potential up to ∼40 mV. At this voltage Na^+^ and Ca^2+^ channels activate, which causes the rapid upstroke of the action potential. Inactivation of the Na^+^ current, and activation of the A-type and the delayed rectifier K^+^ current, as well as the transient, outward part of SGLT1-current, contribute to controlling the peak of the action potential. The delayed rectifier K^+^ current is the major current responsible for repolarization. Note that the ATP-sensitive K^+^ current is relatively big.

In primary L-cells (Fig 5C and 5D) the T-type Ca^2+^ current plays a crucial role in depolarizing the membrane potential, which leads to, first, activation of Na^+^ channels, and, second, activation of HVA Ca^2+^ channels. Inactivation of Na^+^ and T-type Ca^2+^ channels in addition of activation of the delayed rectifier K^+^ current and the transient SGLT1 currents control the action potential amplitude and cause repolarization. The K(ATP)-current is small compared to the other currents.

This insight can explain how changes in *G*_SGLT1_ and *g*_*K*(*ATP*)_ mainly control action potential frequency in our simulations of GLUTag cells, but amplitude in primary L-cells. The effect of *G*_SGLT1_ on *I*_*SGLT*1_ is twofold (Fig 2): it increases the sustained, inward current, but reduced the transient outward current. Since the sustained current is important for depolarization in GLUTag cells, an increase in *G*_SGLT1_ and consequently in the sustained SGLT1 current will reduce the interspike interval, i.e., increase the firing frequency. This effect is not present in primary cells, where the T-type Ca^2+^ currents is playing the main role in the depolarization. Similarly, reduced K(ATP)-channel conductance has a big influence on the interspike interval in GLUTag cells, since the K(ATP)-current is one of the dominant currents between action potentials (Fig 5B). In primary L-cells, changes in the tiny K(ATP)-current does not affect the interspike interval, which is controlled by T-type Ca^2+^ channels (Fig 5D).

In contrast, the transient, outward SGLT1 current is more important for controlling the action potential height in primary cells because of the lack of the A-type K^+^ current. The role of the A-type K^+^ current in GLUTag cells appears to be to control the amplitude of the action potential, since it activates rapidly during the upstroke, and then inactivates (Fig 5B). Reduced K(ATP) channel conductance has a bigger effect on peak voltage in primary L-cells (Fig 4D) than in the GLUTag cell line (Fig 1D) since *I*_*K*(*ATP*)_ is more important during the upstroke in primary L-cells (Fig 5D) compared to GLUTag cells where the A-type K^+^ current activates (Fig 5B). Thus, it is the presence or absence of complimentary currents, notably the A-type K^+^ current and the T-type Ca^2+^ current, that determines the effect of changes in *G*_SGLT1_ and *g*_*K*(*ATP*)_.

### Role of Na^+^ channels

#### GLUTag cells

**Fig 6:**
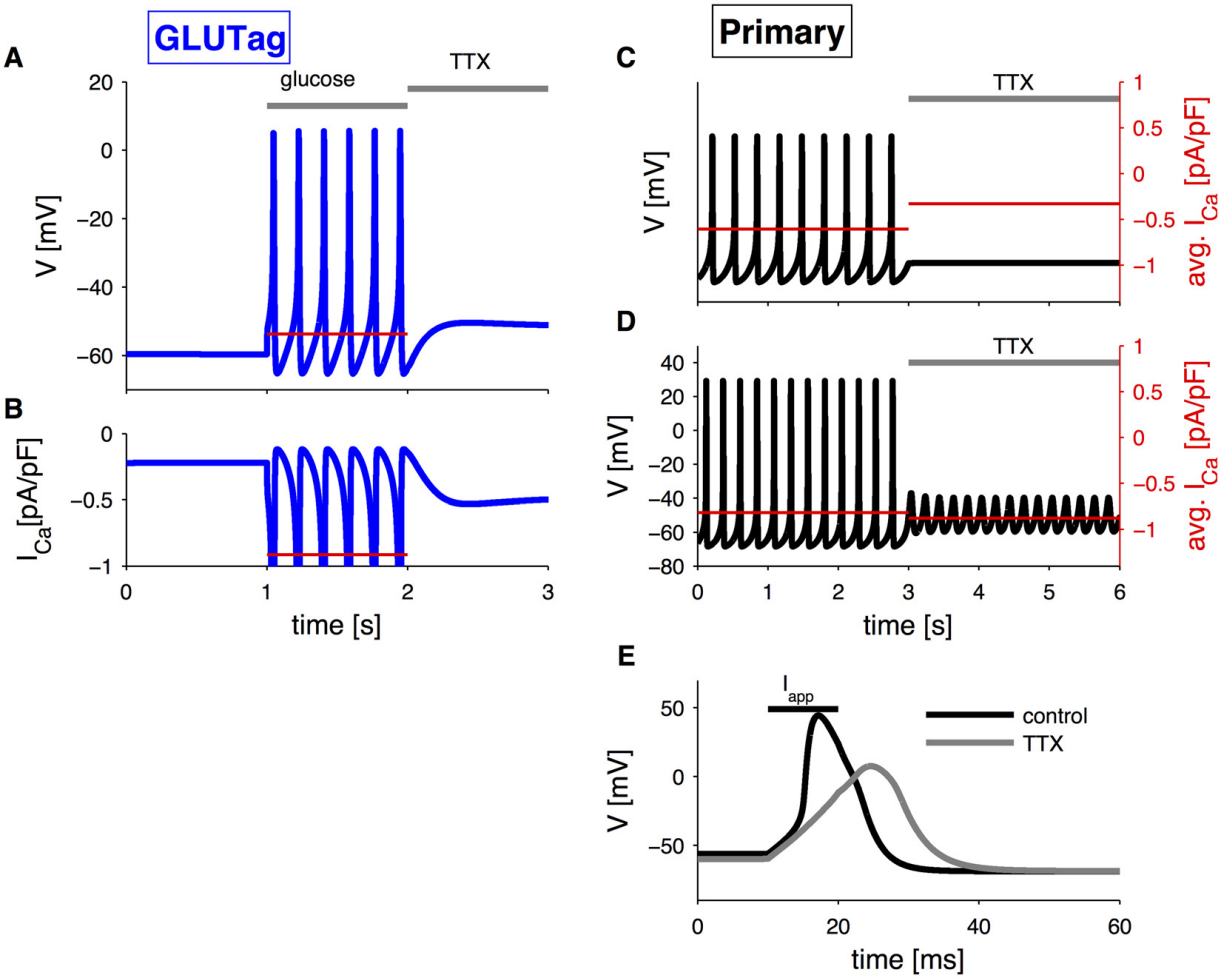
Simulation of application of Na^+^-channel blocker TTX. A: Simulation of glucose-induced electrical activity (*G*_SGLT1_ = 10 mM and *g*_*K*(*ATP*)_ = 0.015 nS/pF) and subsequent application of TTX in GLUTag cells with default parameters. Red line represents mean voltage during electrical activity. B: Calcium current corresponding to the simulation in panel A. Red line represents mean Ca^2+^ current during electrical activity. C: Simulation of current-induced electrical activity (*I*_*app*_ = 0.1 pA/pF, *G*_SGLT1_ = 1 mM and *g*_*K*(*ATP*)_ = 0.0035 nS/pF), and subsequent application of TTX in primary L-cells. The average Ca^2+^ current is indicated by the red lines (right axis) D: As in panel C, except *g*_*CaT*_ = 0.11 nS/pF. E: Simulation of current-evoked action potential in primary L-cells in control case (black line) and in presence of TTX (grey line). Black bar indicates the current application *I*_*app*_ = 5 pA/pF. Grey bars indicate glucose and TTX application as indicated.

Experimentally, the application of the Na^+^-channel blocker TTX in the GLUTag cell line blocks action potentials evoked by 10 mM glucose completely, but did not prevent glucose-triggered rise in intracellular Ca^2+^ and had no effect on GLP-1 secretion [23]. These results are likely due to the ability of glucose to depolarize the cell, both in presence and absence of TTX, which may cause a sustained Ca^2+^current [23]. This explanation could be verified using the model by comparing three conditions: 0.1 mM glucose and no TTX (*G*_SGLT1_ = 0.1 mM and *g*_*K*(*ATP*)_ = 0.03 nS/pF), 10 mM glucose stimulation and no TTX (*G*_SGLT1_ = 10 mM and *g*_*K*(*ATP*)_ = 0.015 nS/pF), and 10 mM glucose stimulation with TTX present (*G*_SGLT1_ = 10 mM, *g*_*K*(*ATP*)_ = 0.015 nS/pF and *g*_*Na*_ = 0 nS/pF). We can directly compare the mean voltage and mean Ca^2+^ current, represented by red lines during electrical activity, in the different conditions (Fig 6A and 6B). Glucose depolarizes the cell by ∼10 mV, both in absence and in presence of TTX. The mean Ca^2+^ current is highest during electrical activity in presence of glucose and absence of TTX. However, while TTX application reduces the current, it is still higher than in the absence of glucose, which might explain why glucose simulates GLP-1 secretion also in the presence of TTX in spite of the absence of action potential firing.

#### Primary L-cells

The important role of Na^+^-channels in the upstroke of the action potentials in primary L-cells was confirmed by the model. We note that GLP-1 release was reduced slightly but statistically significantly by TTX in primary cell cultures [24]. An example of electrophysiological response to TTX was reported by [24]. The primary L-cell, maintained in a depolarized state by continuous injection of a small depolarizing current, fired spontaneous action potentials that were dramatically reduced in frequency, but not completely abolished by TTX. Simulation of Na^+^-channel block in similar conditions, and with default parameters, completely abolished electrical activity and reduced Ca^2+^ influx (Fig 6C). However, given that only one example was shown by Rogers et al. [24] and considering heterogeneity between cells, we further analyzed the model response to TTX, varying the parameters within physiological limits. For example, by increasing T-type Ca^2+^-channel conductance from 0.075 nS/pF to 0.11 nS/pF, TTX application resulted in membrane potential oscillations between -60 mV and -40 mV (Fig 6D), which is close to the threshold for the action potential generation. This simulated oscillatory behavior resembles the fluctuations around baseline observed experimentally [24]. As a consequence, the very low frequency recorded experimentally after TTX application might have been the result of noise, which occasionally allowed the membrane potential to cross the threshold and fire an action potential. Notably, whole-cell Ca^2+^ influx was nearly unchanged in this simulation (Fig 6D).

Furthermore, in presence of TTX, action potentials could still be evoked by current injection, but compared to the control case, they were wider, elicited at a higher threshold and had a smaller amplitude [24]. The model predicts a threshold of ∼-40 mV for initiation of action potentials, which is close to the one found experimentally of ∼-36 mV [24]. Blocking Na^+^-channels in the model, a membrane potential of ∼-10 mV should be reached to activate sufficient Ca^2+^ channels to continue the depolarization trend, even after the applied current is removed (Fig 6E). The decrease in the action potential amplitude caused by TTX is similar (∼40 mV) to the experimental example reported in [24].

### Role of Ca^2+^ channels

#### GLUTag cells

**Fig 7:**
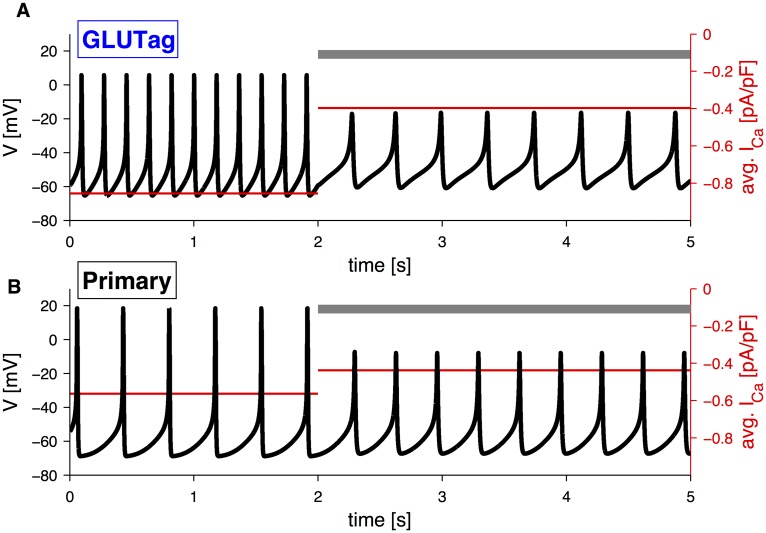
Simulation of application of Ca^+^-channel blocker. A: Simulation of application of the L-type Ca^2+^-channel blocker, nifedipine, in GLUTag cells, with default parameter except *G*_SGLT1_ = 10 mM, *g*_*K*(*ATP*)_ = 0.015 nS/pF. The average Ca^2+^ current is indicated in red (right axis). B: Simulation of application of partial block of HVA Ca^2+^-channels in primary L-cells, with default parameters except *G*_SGLT1_ = 10 mM. The average Ca^2+^ currents are in both panels indicated in red (right axes). Grey bars indicate Ca^2+^-channel blocker application.

The evaluation of the role of Ca^2+^-channels is fundamental, given the association of Ca^2+^ with the exocytotic process. In experiments with GLUTag cells, the application of the L-type calcium channel blocker nifedipine in presence of 10 mM glucose caused a reduction in action potential frequency [23]. The GLUTag mathematical model has a single Ca^2+^ current, and does not differentiate between Ca^2+^ channel types. To simulate the nifedipine effect, Ca^2+^-channel conductance was reduced from 0.24 to 0.14 nS/pF, which is comparable to the barium current inhibition caused by nifedipine application in GLUTag cells [23]. The resulting model simulation showed both a lower peak amplitude and a dramatic reduction in action potential frequency (Fig 7A). As a result, calcium influx was substantially reduced (Fig 7A, red).

#### Primary L-cells

Experimentally, the block of L- or Q-type Ca^2+^-channels in primary L-cell preparations caused a similar and significant reduction in GLP-1 secretion, both under basal and glutamine-stimulated conditions [4]. Simulation of partial block of HVA Ca^2+^-channels, which are a combination of L- and Q-type Ca^2+^-channels, significantly reduced the peak amplitude of glucose stimulated electrical activity, which, in addition to reduced Ca^2+^ influx, might underlie the reduction of secretion found experimentally (Fig 7B) [4].

## Discussion

The relative contribution of SGLT1 and GLUT2 glucose transporters to glucose sensing in the intestinal L-cells has been a matter of debate [8]. Whereas SGLT1 transporters are electrogenic and could promote electrical activity on their own, glucose transported via GLUT2 should be metabolized to increase the ATP/ADP ratio and close K(ATP)-channels, which could lead to action potential firing as in pancreatic *β*-cells. Glucose entering via SGLT1 or GLUT2 could also reduce K(ATP)-channel activity. However, accumulating evidence support the main role of the SGLT1-mediated current in primary L-cells [5–9], whereas both SGLT-1 currents and K(ATP)-channel closure contribute to stimulus-secretion coupling in GLUTag cells [7, 21]. Elevations in intracellular glucose levels could also have effects downstream of electrical activity and Ca^2+^ influx, as has been shown in GLUTag cells [7], and resembling the ‘amplifying pathway’ operating in pancreatic *β*-cells [42].

The theoretical analyses presented here provide new insight into how the electrophysiological differences between primary L-cells and the GLUTag cell line lead to their diverse glucose-sensing mechanisms. The model further suggests that the two cell types encode the glucose signal in electrical activity in different ways: primary L-cells appear to use action potential amplitude (cf. Fig 4D and 4E) to transduce glucose sensing to Ca^2+^ influx and exocytosis, while the model predicts that GLUTag cells rely mainly on changes in firing frequency (Fig 1D and 1E) as found experimentally [22]. We explained this difference by the presence of A-type K^+^ currents in GLUTag cells and T-type Ca^2+^ channels in primary L-cells (cf. Fig 5). Related, we note that small changes in SGLT-1 substrate lead to rapid action potential firing in primary L-cells (Fig 4E) but not in GLUTag cells (Fig 1E). This difference might be related to the lower *α*MG sensitivity in GLUTag cells, which show little secretion to 5 mM *α*MG [21], whereas primary L-cells have a EC_50_-value of ∼0.2 mM for *α*MG-triggered GLP-1 secretion [4]. A limitation of the current version of the model is that it is based on data from cultured cells that have lost their natural polarization, and possibly other characteristics. Future modeling of electrophysiology, Ca^2+^ dynamics and secretion based on mechanistic data from L-cells *in situ* will likely shed further light on the relative importance of action potential amplitude and frequency for GLP-1 secretion.

In the isolated intestine, reflecting the situation *in vivo*, SGLT1 transporters are located on the luminal, apical side of the L-cells, and are therefore exposed directly to glucose in the intestine [8]. In contrast, the GLUT2 transporters are located on the basolateral, vascular side of the L-cells [8], where they allow glucose to pass between the cytosol of L-cells and the plasma. There are reports of GLUT2 protein being transported to and inserted in the luminal membrane of enterocytes in response to glucose entering via SGLT1 [6, 25, 26] (but see [8]). However, even in experiments where luminal GLUT2 expression increased, SGLT1-mediated glucose transport still predominated [6]. GLUT2 knock-out mice have been reported to have ∼50% less GLP-1 release than wild-type animals [43], while another study found unchanged GLP-1 release in GLUT2 knock-out mice [8]. Of note, GLP-1 content is reduced by ∼50% compared to control animals [43], which complicates reasoning on whether GLUT2 plays a role in glucose sensing in L-cells based on studies in GLUT2 knock-out animals. In contrast, luminal GLUT2 inhibition by phloretin has been shown to reduce but not abolish GLP-1 secretion in the perfused rat small intestine [9]. GLUT2 inhibition also abolished a SGLT1-independent component of GLP-1 secretion in isolated loops of small rat intestine [26], but of note this SGLT-1 independent component was not observed in isolated rat small intestine [9] or *in vivo* in mice [5], where the SGLT1 inhibitor phloridzin abolished glucose induced GLP-1 secretion. In summary, there is an ongoing debate of the role of apical GLUT2 in intestinal glucose absorption, which might be due to differences in experimental procedures [8, 25]. Further, whether the mechanisms postulated for enterocytes are operating in L-cells still need to be shown directly. The presented model is unable to provide further insight into these question, mainly since it was build from data from cultured cells; mechanistic experimental results from L-cell *in situ* are needed before we can investigate these questions theoretically.

Interestingly, vascular perfusion with high glucose concentrations in the presence of 3.5 mM luminal glucose triggered GLP-1 secretion in the isolated porcine intestine [44], but in the isolated rat intestine vascular glucose did not lead to GLP-1 release in the absence of luminal glucose [9]. Besides species differences, these conflicting results can be explained as follows. In the presence of 3.5 mM luminal glucose, the SGLT1 current is operating, and vascular glucose can augment GLP-1 secretion by entering the L-cells via basolateral GLUT2 leading to metabolism and closure of K(ATP) channels. In contrast, in the absence of intestinal glucose and SGLT1 current, the closure of K(ATP) channels is insufficient to trigger electrical activity and GLP-1 secretion. In terms of our model, the presence of luminal glucose allows the L-cells to operate further to the right in Fig 4D and 4E where small downward movements due to reduced K(ATP)-conductance more easily lead to electrical activity.

These various experiments point to a mechanism where SGLT1 is the major glucose-sensing component in primary L-cells, but glucose metabolism leading to K(ATP)-channel closure might play a modulating role. The theoretical results presented here support this picture. Pharmacological modulation of K(ATP)-channels can overwrite glucose-sensing, i.e. K(ATP)-channel closure by tolbutamide can trigger electrical activity and secretion in primary L-cells even in the absence of glucose [4, 9, 17], and the K(ATP)-channel agonist diazoxide abolishes glucose-stimulated GLP-1 secretion [9, 24], which can be explained with the model as follows. Pharmacological modification of K(ATP)-channel conductance can push the system in or out or the area with activity, independently of glucose-sensing by SGLT. Such modulation of K(ATP)-channel activity corresponds to large vertical moves in Fig 4D and 4E such that horizontal movements (SGLT1-mediated sensing) are ineffective.

In the basal state the L-cells have a K(ATP)-conductance of <10 pS [4], which corresponds to just a single K(ATP)-channel being open on average [40]. Thus, tolbutamide would have very little K(ATP)-channel conductance to act upon. Nonetheless, our simulations showed that a further reduction in K(ATP)-channel conductance is sufficient to allow electrical activity. In contrast, the GLUTag cells have K(ATP)-channel conductance an order of magnitude larger than the primary L-cells [22]. This fact means that physiological modulation of K(ATP)-channel activity becomes a more reliable glucose-sensing mechanism in the GLUTag cell line, as highlighted by the findings that stimulated metabolism by fructose [21] or glucokinase activators [7] stimulate secretion in GLUTag cells but not in primary L-cells. During an oral glucose tolerance test, tolbutamide does not trigger further GLP-1 release [45]. In this condition, the luminal glucose concentration is high, meaning that the L-cells are active and operating far to the right in Fig 4D and 4E. A reduction in K(ATP)-channel conductance because of tolbutamide application, corresponding to a downward movement in Fig 4D and 4E, will therefore have very little effect. We are unaware of any results showing whether tolbutamide in the absence of ingested glucose stimulates GLP-1 release *in vivo*. However, it has been shown that fructose, which enters via non-electrogenic GLUT5 transporters and most likely act via K(ATP)-channel closure, stimulate GLP-1 secretion in humans *in vivo* [36].

While the role for Na^+^-channels in the generation of action potentials is clear in both GLUTag [23] and primary L-cells [24], their importance for GLP-1 secretion is—surprisingly—less evident. The addition of the Na^+^-channel blocker TTX does not change glucose-stimulated GLP-1 secretion from GLUTag cells [23], while both basal and glutamine-stimulated GLP-1 secretion from primary L-cell cultures are lowered slightly and to the same extent by TTX [24]. Another Na^+^-channel blocker, lidocaine, did not lower glucose-stimulated GLP-1 secretion from perfused rat intestines [9].

Our model simulations showed that, in line with experiments, glucose was able to depolarize GLUTag cells in the presence of TTX, but demonstrated also that the mean Ca^2+^ current was smaller in the presence of TTX than during electrical activity in the absence of TTX (Fig 6). In simulated primary L-cells, Ca^2+^ influx was either reduced or unaffected by TTX, depending on the conditions (Fig 6). If the modest glucose-induced elevation in Ca^2+^ current in the presence of TTX is sufficient to trigger maximal secretion, for example because of depletion of the pool of releasable secretory vesicles, then secretion in presence or absence of TTX would be similar, as seen experimentally in GLUTag cells [23], rather than slightly reduced as reported for cultured primary L-cells [24]. However, this interpretation is at odds with the fact that glucose-evoked Ca^2+^ elevations in GLUTag cells were unaffected by TTX [23]. It might be that the small Ca^2+^ current evokes Ca^2+^-induced Ca^2+^ release (CICR), which then is responsible for triggering exocytosis, suggesting that it is the depolarization of the base-line rather than action potential firing that causes GLP-1 secretion. Interestingly, CICR is an important component of glucose-sensing in pancreatic *δ*-cells [46], and glucose amplifies GLP-1 secretion in GLUTag cells downstream of Ca^2+^ influx [7]. Clearly, the importance of Na^+^ channels and electrical activity in L-cells needs further investigation.

So which of the two cell types investigated here, GLUTag or cultured mouse colonic L-cells, resemble human physiology the most? As human, rat and mouse L-cells *in vivo* [36], GLUTag cells release GLP-1 in response to fructose [21, 36], and GLUT2 inhibition reduce GLP-1 release from these cells [7], similarly to the perfused rat small intestine [9], and isolated loops of small rat intestine [26]. These properties point to a role of K(ATP)-channels and/or metabolism-dependent ‘amplifying pathways’, which augment secretion at a given Ca^2+^ level [7], in GLUTag cells. Thus, in these aspects GLUTag cells surprisingly resemble *in vivo* physiology more than primary cultured L-cells, which are unaffected by GLUT2 inhibition [7] and respond poorly to fructose (personal communication, F. Gribble and F. Reimann, University of Cambridge, U.K.). However, cultured primary mouse L-cells clearly depend more strongly than GLUTag cells on SGLT1, since the SGLT1 blocker phloridzin virtually abolish GLP-1 secretion from primary cell cultures, but only lowers release from GLUTag cells by 40–50% [7, 21]. This strong dependence of SGLT1 in primary L-cell cultures resembles more physiological settings [5, 6, 9]. In summary, cultured primary L-cells are preferable for investigations on SGLT1 and electrophysiology, whereas GLUTag cells appear more similar to *in vivo* physiology with respect to metabolism. Hopefully improvements in isolation and culture procedures, and advanced studies on L-cells *in situ* will allow investigations on primary cells with maintained physiological characteristics.

The model presented here should be valuable also for understanding glutamine-stimulated GLP-1 secretion, since glutamine is co-transported with Na^+^ by the electrogenic glutamine co-transporter [47], and the stimulus pathway is therefore similar to glucose-sensing by SGLT1 investigated here. Further developments of the model will take into account the spatial organization of L-cells, in particular the role of SGLT1 co-transport in the apical membrane in contrast to GLUT2 transport at the basolateral membrane, where GLP-1 is also secreted. Inclusion of GLP-1 vesicle dynamics and stimulation by proteins and fat will also be interesting to study based on the present model as new data emerges. In this context, experiments on primary L-cells, preferably *in situ* with their polarization preserved will in our opinion be necessary to provide further insight. Mathematical modeling can and should be used in interpreting such more physiological data, which in turn will guide the evolution of the model developed here.

## Methods

### Modeling of electrical activity

A single mathematical Hodgkin-Huxley-type model that, depending on the parameters, describes electrical activity in primary mouse L-cells or in the GLP-1 secreting cell line GLUTag [20] was developed. The model and the two parameter sets were based on patch clamp data from primary colonic L-cells [24] and GLUTag cells [23], respectively. The model includes ATP-sensitive K^+^-channels (K(ATP)-channels), voltage-gated Na^+^-, K^+^- and Ca^2+^-channels, and the electrogenic sodium glucose co-transporter SGLT1.

The evolution of the membrane potential *V* is driven by the contribution from the different currents (normalized by cell capacitance) to be described in details below,

$$\frac{dV}{dt}=-\left(I_{Na}+I_{CaT}+I_{CaHV\;A}+I_{Kv}+I_{KA}+I_{K,hyper}+I_{SGLT}+I_{K(ATP)}\right).$$ (1)

Voltage-gated membrane currents are modelled as

$$I_{X}=g_{X}m_{X}h_{X}\left(V-V_{X}\right),$$ (2)

where *X* stands for the channel type, *V*_*X*_ is the associated reversal potential, *g*_*X*_ the maximal whole-cell channel conductance, and *m*_*X*_ and *h*_*X*_ describe activation and inactivation of the channel, respectively.

Activation (similarly inactivation) is described by

$$\frac{dm}{dt}=\frac{m_{X,\infty }\left(V\right)-m_{X}}{\tau_{mX}},$$ (3)

where *m*_*X*, ∞_ is the steady-state voltage-dependent activation function, and *τ*_*mX*_ is the time-constant of activation, which in some cases depends on the membrane potential. Steady-state voltage-dependent activation (inactivation) functions were described by the Boltzmann equation

$$m_{X,\infty }=\frac{1}{1+e^{\left(V-V_{mX}\right)/k_{mX}}}.$$ (4)

Reimann et al. [23] reported non-normalized currents for GLUTag cells. In order to normalize these currents, we estimated the cell capacitance *C* to be ∼7 pF from the results by Gribble et al. [21], who reported that 100 mM alpha-methyl-D-glucopyranoside (*α*MG) induced a current of ∼5 pA/cell or ∼0.7 pA/pF in GLUTag cells.

Parameters for membrane currents can be found in Table 1, whereas parameters for the SGLT1 model are given in Table 2. Simulations were performed in XPPAUT [48] with the cvode solver. Computer code can be found in the Supplementary Material.

**Table 1:** Default parameters of the different ion channels.

Parameter	Primary	Ref	GLUTag	Ref	Unit
*V*_*Na*_	69		69		mV
*g*_*Na*_	2.1	[24]	1.7	*[23]*	nS/pF
*V*_*mNa*_	-19	[24]	-9.5	*[23]*	mV
*k*_*mNa*_	-5	[24]	-6.7	*[23]*	mV
*V*_*hNa*_	-46	[24]	-39.7	[23]	mV
*k*_*hNa*_	6	[24]	9.2	[23]	mV
*τ*_*hNa*_	3	*[24]*	1.5	*[23]*	ms
*V*_*Ca*_	65		65		mV
*g*_*CaHV A*_	0.29	*[24]*	0.24	*[23]*	nS/pF
*V*_*mCaHV A*_	-5	[24]	-13.7	*[23]*	mV
*k*_*mCaHV A*_	-6	[24]	-9.4	*[23]*	mV
*V*_*hCaHV A*_	-23	*[24]*	-25	[23]	mV
*k*_*hCaHV A*_	13	*[24]*	8.5	[23]	mV
*τ*_*hCaHV A*_	100	*[24]*	40	*[23]*	ms
*A*	0.38	*[24]*	0.5	*[23]*	ms
*g*_*CaT*_	0.075	*[24]*	0	[23, 24]	nS/pF
*V*_*mCaT*_	-40	[24]	-		mV
*k*_*mCaT*_	-7	[24]	-		mV
*V*_*hCaT*_	-62	*[24]*	-		mV
*k*_*hCaT*_	20	*[24]*	-		mV
*τ*_*hCaT*_	20	*[24]*	-		ms
*V*_*K*_	-70		-70		mV
*g*_*K*_	2.5	[24]	1.7	*[23]*	nS/pF
*V*_*mK*_	5.2	[24]	0.5	[23]	mV
*k*_*mK*_	-15	[24]	-11.2	[23]	mV
*τ*_0_	30	*[24]*	10	*[23]*	ms
*τ*_1_	40	*[24]*	20	*[23]*	ms
*V*_*τ*_	20	*[24]*	10	*[23]*	mV
*k*_*τ*_	5	*[24]*	10	*[23]*	mV
*g*_*A*_	0		0.65	*[23]*	nS/pF
*V*_*mA*_	-		3.9	*[23]*	mV
*k*_*mA*_	-		-23.5	*[23]*	mV
*V*_*hA*_	-		-61	[23]	mV
*k*_*hA*_	-		7.5	[23]	mV
*τ*_*hA*_	-		30	*[23]*	ms
*g*_*Hyper*_	0		0.1	[23]	nS/pF
*V*_*Hyper*_	-		-40.2	[23]	mV
*V*_*mHyper*_	-		-85.7	[23]	mV
*k*_*mHyper*_	-		9.2	[23]	mV
*τ*_*mHyper*_	-		500	*[23]*	ms
*g*_*K*(*ATP*)_	0.003	[4]	0.03	*[22]*	nS/pF

References in italic indicate that the parameter is obtained by fitting data reported in the citation.

**Table 2:** Default parameters of the SGLT1 model.

Parameter	Primary	Ref.	GLUTag	Ref.	Unit
*n*	4e6		7.7e6	[22]	adim
*C*	8	[24]	7	[21]	pF
$k_{12}^{0}$	8e-5	[49]	8e-5	[49]	ms^−1^ mM^−2^
$k_{21}^{0}$	0.5	[49]	0.5	[49]	ms^−1^
*α*	0.3	[49]	0.3	[49]	adim
*k*_23_	0.1	[49]	0.1	[49]	ms^−1^mM^−1^
*k*_32_	0.02	[49]	0.02	[49]	ms^−1^
*k*_25_	3e-4	[49]	3e-4	[49]	ms^−1^
*k*_52_	3e-4	[49]	3e-4	[49]	ms^−1^
*k*_34_	0.05	[49]	0.05	[49]	ms^−1^
*k*_43_	0.05	[49]	0.05	[49]	ms^−1^
*k*_45_	0.8	[49]	0.8	[49]	ms^−1^
*k*_54_	40	[49]	40	[49]	ms^−1^mM^−1^
*k*_56_	0.01	[49]	0.01	[49]	ms^−1^
*k*_65_	5e-8	[49]	5e-8	[49]	ms^−1^mM^−2^
$k_{16}^{0}$	0.035	[49]	0.035	[49]	ms^−1^
$k_{61}^{0}$	5e-3	[49]	5e-3	[49]	ms^−1^
*δ*	0.7	[49]	0.7	[49]	adim

#### Voltage-gated sodium-channels

In GLUTag cells, voltage steps triggered rapidly inactivating Na^+^ currents (*I*_*Na*_) at potentials higher than -40 mV. Inactivation parameters were used from [23] without modification, with an estimated time constant of 1.5 ms based on reported voltage clamp traces. Channel conductance and activation function were obtained from fits to the current-voltage (I-V) relationship [23]. Given the rapid activation, Na^+^-channels were assumed to activate instantaneously, i.e., *m*_*Na*_ = *m*_*Na*, ∞_(*V*).

**Fig 8:**
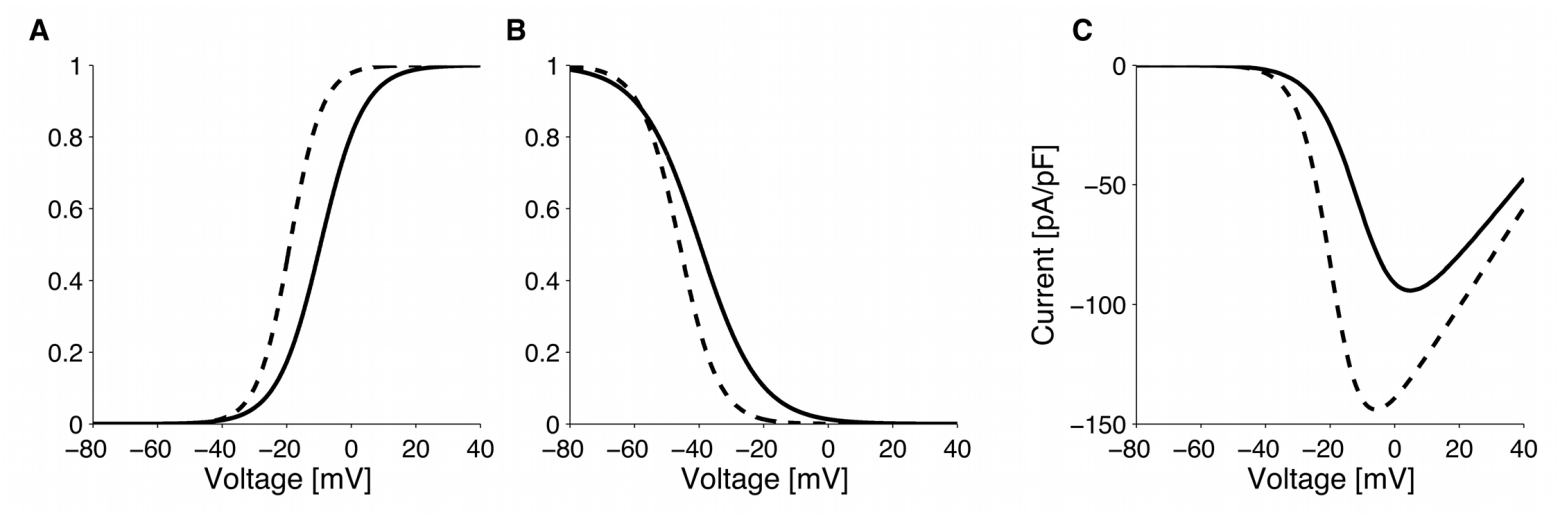
Sodium currents. Comparison of Na^+^ current (*I*_*Na*_) activation function (A), inactivation function (B) and I-V relationship (C) between GLUTag (solid line) and primary L-cells (dashed line).

In primary murine L-cells, Na^+^ currents were reported to have fast activation and to undergo large and rapid inactivation. Hence, they were assumed to activate instantaneously, while the inactivation kinetics was estimated by simulating voltage clamp experiments [24] (*τ*_*mNa*_ ≈ 3 ms). Activation and inactivation parameters were used without modification from [24]. The conductance value was slightly (∼10%) increased compared to the value reported by Rogers et al. [24], but the resulting I-V relationship was within experimental error bars [24]. Compared to GLUTag cells, primary L-cells have a bigger sodium current with activation function left-shifted by ∼10 mV (Fig 8).

#### Voltage-activated calcium-channels

The calcium I-V relationship in GLUTag cells exhibit a single peak [23], probably due to the lack of the low voltage activated T-type Ca^2+^-channels in the cell line [24]. Hence, the Ca^2+^ current in GLUTag cells was modelled as a single high-voltage-activated (HVA) current (*I*_*CaT*_ = 0 pA/pF in Eq (1)). Since half of the current inactivates [23], the inactivation function was modelled as

$$h_{CaHV\;A,\infty }=\left(1-A\right)+Ah_{CaHV\;A,\infty }^{*},$$ (5)

with *A* = 0.5 and $h_{CaHV\;A,\infty }^{*}$ as in Eq (4). Inactivation parameters were used without modification from [23] and an estimated time constant of ∼40 ms compatible with voltage clamp experiments. Channel conductance and activation function were obtain from the I-V relationship reported by Reimann et al. [23].

**Fig 9:**
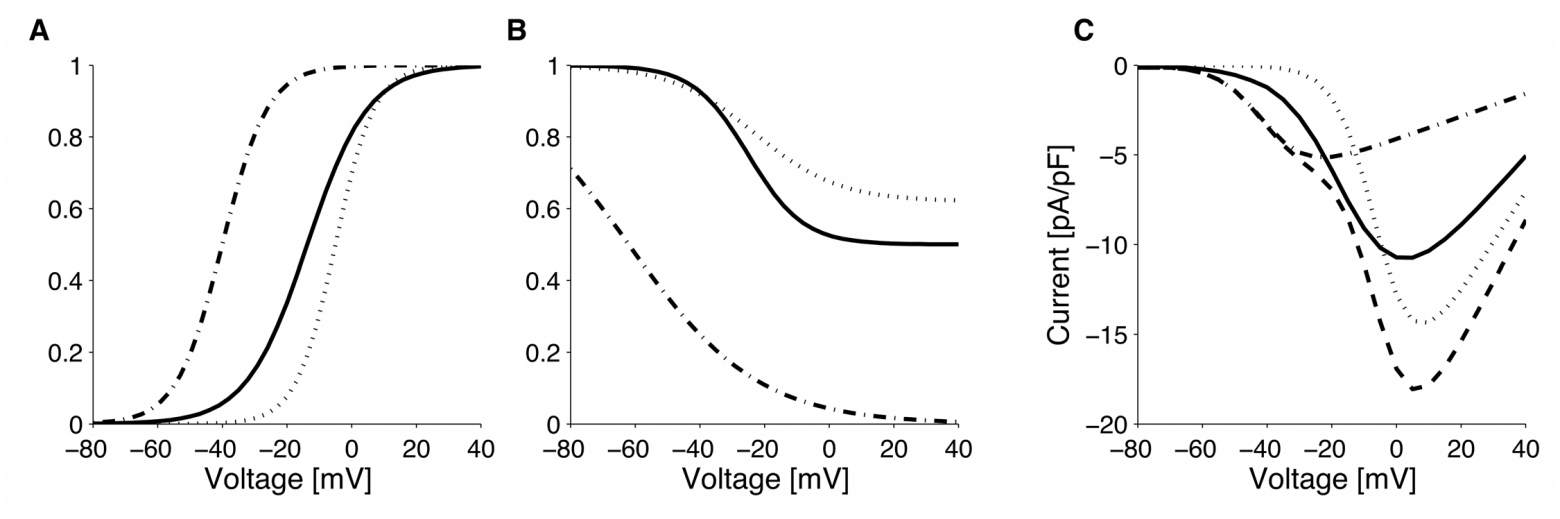
Calcium currents. A: Comparison of HVA Ca^2+^ current (*I*_*CaHVA*_) activation function between GLUTag (solid line) and primary L-cells (dotted line), superimposed T-type Ca^2+^ current activation function (dot-dashed line). B: Comparison of HVA Ca^2+^ current (*I*_*CaHVA*_) inactivation function between GLUTag (solid line) and primary L-cells (dotted line), superimposed T-type Ca^2+^ current inactivation function (dot-dashed line). C: Comparison of HVA Ca^2+^ current (*I*_*CaHV A*_) -V relationship between GLUTag (solid line) and primary L-cells (dotted line), superimposed T-type Ca^2+^ current activation function (dot-dashed line) and the total Ca^2+^ current (dashed line) in primary L-cells.

In primary murine L-cells, Ca^2+^-currents have fast activation and approximately half of the total Ca^2+^ current inactivates. Moreover, voltage dependence of the peak current presents a clear shoulder around −30 mV, suggesting the presence of low- (T-type, *I*_*CaT*_) and high- (L- and Q-type, *I*_*CaHV A*_) voltage-activated Ca^2+^ currents [24]. We assumed instantaneous activation of the Ca^2+^-currents. Overall, the primary L-cells have larger Ca^2+^ currents than the GLUTag cells (Fig 9).

The T-type Ca^2+^-channels were assumed to inactivate completely. Accordingly the I-V relationship for the steady state Ca^2+^-current lacks the shoulder around −30 mV of the peak current [24]. The high voltage activated Ca^2+^-channels likely correspond to a combination of L-type and Q-type [24], which in neuronal cells have similar inactivation kinetics [49]. The Ca^2+^-current inactivated to a similar degree in barium and calcium [24], suggesting that inactivation was voltage-dependent. Given the complete inactivation of T-type Ca^2+^-channels, the residual current is due to HVA Ca^2+^-channels only. As a consequence their inactivation function was modelled as in Eq (5), with *A* = 0.38 and $h_{CaHV\;A,\infty }^{*}$ estimated as described below.

To differentiate between inactivation of low- and high-voltage-activated Ca^2+^-channels, we fitted all parameters simultaneously (activation, inactivation and conductance) by least-square optimization using the I-V relationship for peak and steady state Ca^2+^-current along with the overall inactivation function reported by Rogers et al. [24]. As initial values for the fitting procedure, we used the activation parameters and conductances reported in [24] supplemented with reasonable guesses for inactivation parameters. The activation parameters and conductances resulting from the fitting procedure were in good agreement with the values found by Rogers et al. [24]. Inactivation of Ca^2+^-currents in response to voltage steps evoking maximal peak current, i.e., where HVA Ca^2+^-channels represent the dominant component, is slower than at less depolarized pulses [24]. Consequently, we assumed that T-type Ca^2+^-channels inactivation was faster (∼20 ms) than HVA Ca^2+^-channel inactivation (∼100 ms).

#### Voltage-dependent potassium-channels

**Fig 10:**
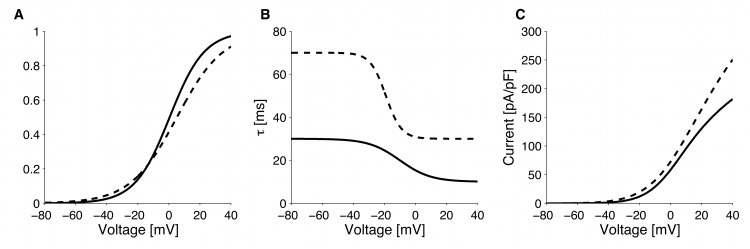
Potassium currents. Comparison of delayed-rectifier K^+^ current (*I*_*Kv*_) activation function (A), time constant (B) and I-V relationship (C) between GLUTag (solid line) and primary L-cells (dashed line).

Reimann et al. [23] reported a detailed characterization of K^+^-channels in GLUTag cells. Depolarization steps resulted in voltage-dependent currents, which showed partial inactivation. The dominant component of this current was virtually non-inactivating and TEA-sensitive (*I*_*Kv*_). This current was assumed not to inactivate and to activate with kinetics modelled as

$$\tau_{mKv}=\tau_{0}+\frac{\tau_{1}}{1+e^{\left(\left(V+V_{\tau }\right)/k_{\tau }\right)}},$$ (6)

compatible with voltage clamp experiments. Activation function parameters were taken from [23] without modification, while conductance was estimated from the data [23], as reproduced in Fig 10.

The TEA-insensitive current was reported to have the characteristics of an A-type K^+^ current (*I*_*KA*_), and was assumed to activate instantaneously [23]. Inactivation parameters were used from [23] without modification, inactivation time constant was based on reported voltage-clamp traces, while conductance and activation function were obtain from the experimental I-V relationship [23].

In response to hyperpolarizing voltage steps, a time- and voltage-dependent, non-inactivating current (*I*_*K*,*hyper*_) was observed [23]. Current characterization was taken from [23], with an estimated activation time constant of 500 ms based on voltage-clamp experiments [23].

In the primary murine L-cells, K^+^-currents (mainly delayed-rectifiers, *I*_*Kv*_) exhibit voltage-dependent activation kinetics, which was modelled similarly to Eq (6), but with different parameters values corresponding to experimental traces (Fig 10B) [24].

K^+^-channels in primary L-cells were assumed not to inactivate, given their slow inactivation [24]. Activation parameters were used from [24] without modification, while conductance was slightly (∼10%) decreased compared to the value reported in [24], but the resulting I-V relationship was within error bars.

#### ATP-sensitive potassium-channels

The ATP-sensitive potassium (K(ATP)-) current was modelled as

$$I_{K(ATP)}=g_{K(ATP)}\left(V-V_{K}\right),$$

where *g*_*K*(*ATP*)_ represents the conductance of the ATP-sensitive potassium channels and varies according to the fraction of open channels.

A tolbutamide-sensitive K(ATP)-current was detected in the GLUTag cell line [22]. Its amplitude was estimated from the immediate whole-cell current of ∼0.6 pA/pF in response to 20 mV pulses from −70 mV. Assuming a potassium reversal potential of *V*_*K*_ = −70 mV, we obtain a conductance of *g*_*K*(*ATP*)_ = 30 pS/pF in the GLUTag cells.

Similarly, functional K(ATP)-channels in primary L-cells were identified by the presence of a tolbutamide-sensitive current [4]. The endogenous K(ATP) conductance was estimated from the measured slope conductance just after whole-cell mode was realized [4]. The obtained value was *g*_*K*(*ATP*)_∼3 pS/pF. Thus, the ATP-sensitive potassium current resulted to be an order of magnitude bigger in GLUTag compared to primary L-cells.

#### Sodium/glucose co-transporter model

The sodium/glucose co-transporter 1 (SGLT1) utilizes a concentration gradient of Na^+^ to transport glucose from the intestine into the L-cells. Experimental measurements showed that two Na^+^ ions are required to transport one molecule of glucose into the cell. Moreover, in absence of sodium in the external medium, glucose is not transported [50].

**Fig 11:**
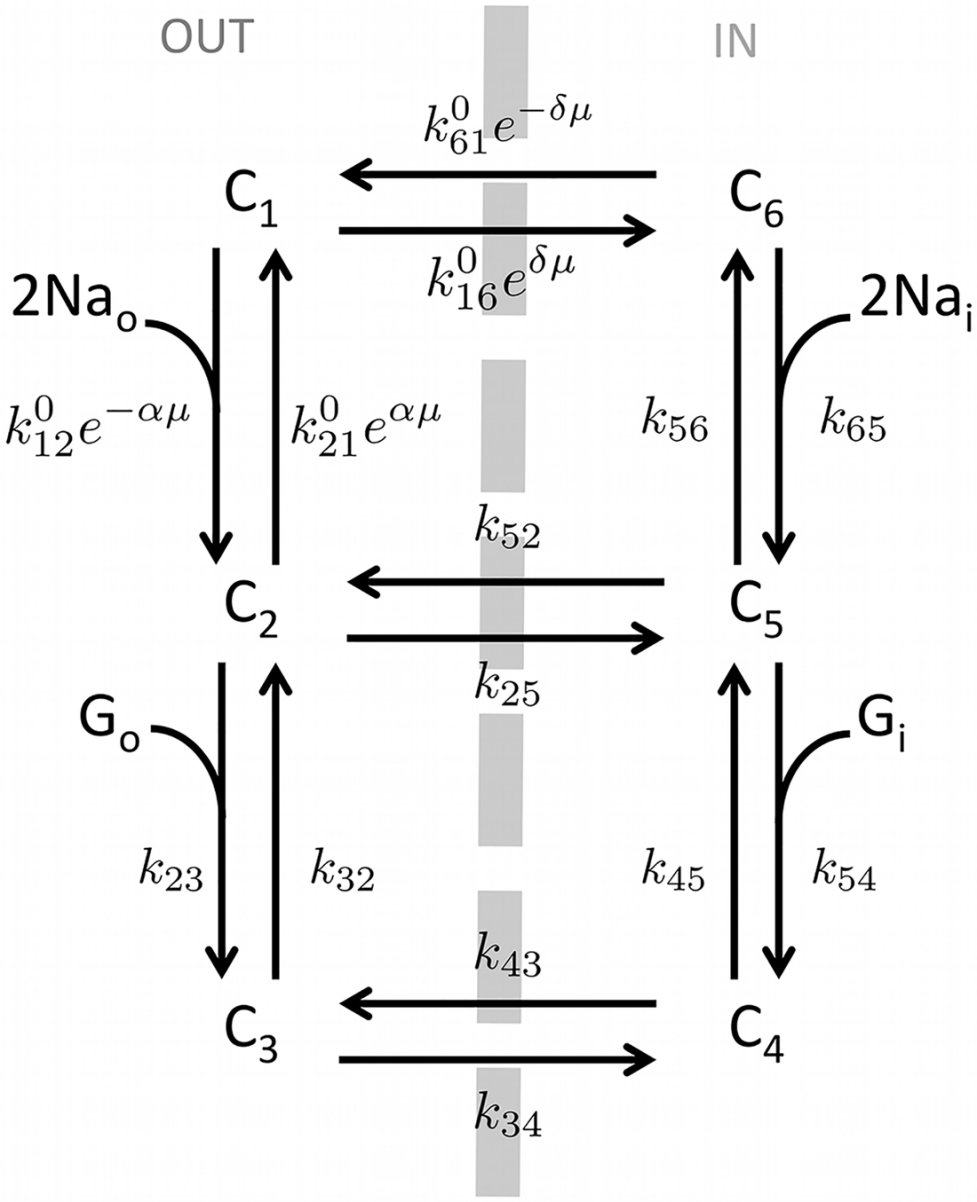
Six-state model of the sodium/glucose co-transporter SGLT1.

These observations lead to a six-state model (Fig 11) [51]. Starting with the empty carrier outside the cell (state 1), the first step is the association of two sodium ions with the carrier (state 2), which allows the subsequent association of glucose (state 3). The third step corresponds to the translocation of the carrier from outside to inside the cell (state 4). Symmetrical steps take place inside the cell consisting in successive dissociation of glucose (state 5) and sodium (state 6). A final step brings the empty carrier back to the initial state outside the cell. The values of rate constants for the six-state model were assigned by [51], and are given in Table 2.

By defining

$$k_{12}=k_{12}^{0}{\left[\text{Na}\right]}_{0}^{2}e^{-\alpha \mu },$$ (7)

$$k_{21}=k_{21}^{0}e^{\alpha \mu },$$ (8)

$$k_{16}=k_{16}^{0}e^{\delta \mu },$$ (9)

$$k_{61}=k_{61}^{0}e^{-\delta \mu },$$ (10)

the small current associated with sodium/glucose co-transport and attributable to the translocation of the negatively charged carrier [51], is given by

$$I_{SGLT}=-\frac{2F}{C}\frac{n}{N_{A}}\left[\alpha \left(k_{12}C_{1}-k_{21}C_{2}\right)+\delta \left(k_{61}C_{6}-k_{16}C_{1}\right)\right],$$ (11)

where *F* is the Faraday constant, *C* the cell capacitance, *n* the number of transporters, *N*_*A*_ the Avogadro’s number, *k*_*xy*_ is the rate constant describing the transition between state *x* and state *y*, *C*_*z*_ is the fraction of carriers in state *z*, and *α* and *δ* are phenomenological coefficients representing fractional dielectric distances. Finally, *μ* is the reduced potential *FV*/*RT*, where *R* is the gas constant and *T* is the temperature.

At steady-state, Eq (11) becomes

$$I_{SGLT}=-\frac{2F}{C}\frac{n}{N_{A}}Flux,$$ (12)

where *Flux* denotes the steady-state translocation flux

$$Flux=k_{12}C_{1}-k_{21}C_{2}=k_{61}C_{6}-k_{16}C_{1}.$$ (13)

The SGLT1 current depends on glucose and sodium concentrations inside and outside the cell, as well as on the membrane voltage *V*, because of the dependence of the rate constants on these factors. Simulated depolarization steps cause an outward transient current and an inward steady state current (see Results).

The magnitude of the SGLT1 current is directly proportional to the number of transporters *n* in the cell. In GLUTag cells, the SGLT1 current was calibrated using the change in the holding current at −70 mV, when *α*MG was applied at saturating concentration (20 mM) [21], which led to *n* = 7.7 × 10^6^, assuming a cell capacitance of *C* = 7 pF [21]. No corresponding data are available for the primary murine L-cells. We assumed that the SGLT1 current was smaller in primary compared to GLUTag cells, similarly to the difference in K(ATP) conductance, since the balance between the SGLT1 and K(ATP) currents determines whether the cell is excitable. These currents should therefore be of comparable magnitude in order to allow the cell to switch from quiescence to action potential firing and vice versa. For the primary murine L-cells, which have cell capacitance *C* ∼ 8 pF [24], we used *n* = 4 × 10^6^.

## Supporting Information

S1 Computer code Computer code for XPPAUT of the model with primary L-cell parameters. (ODE)

S2 Computer code Computer code for XPPAUT of the model with GLUTag cell parameters. (ODE)
